# A Reduction in Pain Intensity Is More Strongly Associated With Improved Physical Functioning in Frustration Tolerant Individuals: A Longitudinal Moderation Study in Chronic Pain Patients

**DOI:** 10.3389/fpsyg.2019.00907

**Published:** 2019-04-26

**Authors:** Carlos Suso-Ribera, Laura Camacho-Guerrero, Jorge Osma, Santiago Suso-Vergara, David Gallardo-Pujol

**Affiliations:** ^1^Department of Basic and Clinical Psychology and Psychobiology, Universitat Jaume I, Castellón de la Plana, Spain; ^2^Hospital Universitario de La Plana, Castellón de la Plana, Spain; ^3^Department of Psychology and Sociology, Instituto de Investigación Sanitaria de Aragón, Universidad de Zaragoza, Teruel, Spain; ^4^Department of Traumatology, Hospital Clinic, Barcelona, Spain; ^5^Department of Clinical Psychology and Psychobiology, Universitat de Barcelona, Barcelona, Spain

**Keywords:** chronic pain, physical performance, longitudinal studies, frustration tolerance, moderator variables, personalized medicine, thinking skills

## Abstract

**Objective:**

The onset and chronification of pain often has devastating consequences on the physical and mental functioning of individuals. Medical interventions are quite efficacious in reducing pain levels. However, changes in physical and mental health status after medical interventions are not proportional. In the past decades, rational/irrational beliefs, especially catastrophizing, have contributed to a better understanding of the pain experience. This study explores whether pain reduction efforts are more beneficial for individuals scoring high in rational thinking (moderation).

**Methods:**

The study design was longitudinal. Patients were assessed twice, 2 weeks prior to the start of medical treatment at the pain clinic and 6 months after. A total of 163 patients with heterogeneous pain (mostly low back and neck pain) participated in the study. Their mean age was 58.74 years (*SD* = 14.28) and 61.3% were female.

**Results:**

Overall, there was a reduction in pain intensity (*t* = 4.25, *p* < 0.001, *d* = 0.32). An improvement in physical functioning (*t* = 4.02, *p* < 0.001, *d* = 0.19), but not mental health (*t* = -0.66, *p* = 0.511, *d* = 0.11) was also observed. In the regression analyses, a decrease in pain intensity was moderately associated with improved physical health (β = 0.87, *t* = 4.96, *p* < 0.001, *R*^2^ change = 0.177). This association was found to be moderated by frustration tolerance (β = -0.49, *t* = -2.80, *p* = 0.006, *R*^2^ change = 0.039). Specifically, *post hoc* analyses indicated that changes in pain intensity only correlated with changes in physical health when patients reported high frustration tolerance levels (*r* = 0.47, *p* = 0.006, *M* = 7, *n* = 32), but not when patients were intolerant to frustration (*r* = 0.28, *p* = 0.078, *M* = 17, *n* = 41).

**Conclusion:**

The results suggest that frustration tolerance may render adaptive by facilitating the positive effect that a reduction in pain intensity has on physical health status. The study findings are discussed in the context of personalized therapy with an emphasis on how to maximize the effectiveness of current interventions for pain.

## Introduction

The onset and chronification of pain in previously healthy individuals often has profound and pervasive effects on the people’s ability to perform physically, as well as on their overall mental well-being (Mehta et al., 2016; Rayner et al., 2016). Not surprisingly, with estimates of chronic pain ranging from 20 to 30% globally (Fayaz et al., 2016; Chenaf et al., 2018), this disease has become one the leading cause of physical disability and sick leave both in Europe and the United States (Gaskin and Richard, 2012; Breivik et al., 2013).

Medical treatment (i.e., oral drugs and surgery) is the most frequent approach in the management of chronic pain (Turk et al., 2011; Gatchel et al., 2014; Reid et al., 2015), as well as the first-line intervention in pain guidelines (Koes et al., 2010; Sarzi-Puttini et al., 2012; Dowell et al., 2016). While there is evidence to support that medical treatment is effective for pain management (Turk et al., 2011; Finnerup et al., 2015), studies have also indicated that such reductions in pain intensity do not lead to a proportional improvement of physical and mental functioning (Gauthier et al., 2008; Menezes Costa et al., 2011; Bendayan et al., 2017), so it is possible that underlying mechanisms that have shown to contribute to a better understanding of the experience of chronic pain (i.e., psychological factors) might as well influence this relationship.

Consistent with the previous idea, research in the past decades has shown psychological factors clearly contribute to a better understanding of the experience of chronic pain (Edwards et al., 2016; Linton et al., 2018; Serrano-Ibáñez et al., 2018). For instance, catastrophizing, a maladaptive form of thinking characterized by a tendency to exaggerate, worry, and anticipate the worst possible consequences of an event (Leung, 2012; Ramírez-Maestre et al., 2017), has been consistently associated with poorer health status of pain patients across numerous investigations (Vancleef and Peters, 2006; Burns et al., 2015; Fallon et al., 2015; Ramírez-Maestre et al., 2017) and has become a fundamental outcome in pain research (Williams et al., 2013). In fact, important for the present investigation, there is previous evidence suggesting an interaction between pain catastrophizing and pain intensity in their relationship with physical health (Suso-Ribera et al., 2017), thus supporting the idea that an interplay between psychological factors and pain intensity in the prediction of health status exists. The extent to which this interplay also occurs longitudinally (i.e., in the relationship between changes in pain intensity and changes in health status) and whether psychological factors other than pain catastrophizing can also act as moderators of pain-health associations remains unclear.

In relation to the latter, an increasing number of psychological factors are now gaining ground in the pain literature, including pain acceptance, coping, self-efficacy, and injustice (McCracken and Eccleston, 2003; Okifuji and Turk, 2015; Yakobov et al., 2018), among others, which suggests that there might be other potential moderators of the pain-to-health relationship in the pain literature. Also importantly, additional forms of irrational/rational thinking other than catastrophizing (Ellis, 1962), namely, demandingness (i.e., rigid requirements expressed in terms of “musts” and “shoulds”), low frustration tolerance (i.e., evaluating certain circumstances as unbearable), and self-downing (i.e., a tendency to make global negative self-evaluations) are starting to receive attention in pain research. For instance, demandingness, in the form of perfectionism, has been associated with higher pain interference and more negative affect in past research (Hadjistavropoulos et al., 2007). Similarly, low frustration tolerance, which has been argued to bear similarities with low acceptance, and self-downing have been associated with poorer mental health status in past research (Suso-Ribera et al., 2016), while self-downing has also shown to contribute to poorer physical functioning (i.e., activity level) when accounting for the role of other pain-related beliefs, such as the belief in the permanence of pain or the tendency self-blame about pain (Stroud et al., 2000). While the aforementioned findings are encouraging and evidence the important role of psychological factors in the pain experience, the extent to which pain catastrophizing and other forms of thinking can help understand why changes in pain intensity after medical treatment are not necessarily associated with improved functioning remains uncertain.

To shed new light into the aforementioned gap in the literature, this study will investigate the moderating role of the previous forms of thinking, namely demandingness, catastrophizing, frustration intolerance, and self-downing, in the relationship between changes in pain intensity and changes in physical and mental health status in a sample of chronic pain patients following a medical intervention. We hypothesize that improvements in health status after a reduction in pain intensity will be facilitated when individuals score high in rational thinking. Conversely, we expect that irrational thinking will result in an inhibition of the positive effects of pain reduction efforts on adaptation to pain (i.e., moderation). These hypotheses come from studies showing that irrational beliefs, which are defined as a maladaptive appraisal of events in which assumptions about reality are inconsistent with that reality, act as underlying cognitive vulnerability factors for distress in front of negative situations, such as experiencing chronic pain (Vîsla et al., 2016; Buschmann et al., 2018). By contrast, rational thinking, which would be characterized by a realistic anticipation and preoccupation about future outcomes (i.e., low catastrophizing), a flexible relationship with the reality in terms of preferences as opposed to demands (i.e., low demandingness), openness to difficult experiences while attempting to reach personal goals (i.e., high frustration tolerance), and a tendency to be self-compassionate and to unconditionally self-accept oneself (i.e., low self-downing), is argued to be in accordance with reality (Vîsla et al., 2016) and, therefore, it would provide resilient resources for well-being (Cristea et al., 2013; Suso-Ribera et al., 2016). In fact, the promotion of rational thinking is a key treatment goal of Cognitive-Behavior Therapy (CBT), perhaps the most popular and empirically supported psychological approaches to a wide range of health problems, including chronic pain (Cristea et al., 2015). In sum, with the present study we expect to find psychological characteristics in the patient that positively influence the pain reduction to pain adaptation relationship.

## Materials and Methods

### Participants

A total of 163 chronic pain patients with non-cancer, musculoskeletal pain participated in this study. All patients were adults aged eighteen or over. Their mean age was 58.74 years (*SD* = 14.28) and 62.0% of them were female. Almost half of patients had not completed secondary education (49.7%), while a smaller percentage had finished technical or university studies (25.8%). At the time of assessment, 36.2% of patients were working, 11.0% were unemployed, and 52.8% were retired.

Duration of pain prior to intervention ranged from 6 months to 49 years, with a median of 2 years (mean = 5.30, *SD* = 7.56). The main pain locations were the lower back (63.9%) and the neck (11.0%). The remaining pain locations occurred at very low frequencies and are not reported to facilitate the readability of the manuscript. Ethnic characteristics were not explored in this study due to the homogeneity of the sample, which was mostly Caucasian. The large majority of participants (93.9%) were Spanish.

### Instruments

#### Pain Intensity

A numerical rating scale (NRS) was used to measure patients’ pain intensity at the time of assessment, with patients being asked to rate their pain intensity from 0 = *no pain* to 10 = *worst possible pain*. Numerical rating scales are the gold standard in the measurement of pain and they are recommended due to their associated compliance rate, responsiveness, and ease of use (Hjermstad et al., 2011).

#### Health Status

The Spanish form of the Short Form-36 Health Survey (SF-36; Ware and Sherbourne, 1992) was administered to evaluate the pain patient’s physical and mental health status. The 36 items in the SF-36 can be grouped into eight dimensions of health, which are either related to physical health (i.e., physical functioning at daily activities, performance at work, pain intensity, and general health) or mental health (i.e., vitality, social functioning, influence of emotions on functioning, and psychological well-being). Two composite scores can be calculated from these eight factors to obtain a Physical Composite Score (PCS) and a Mental Composite Score (MCS). The use of these two broader constructs is preferred as it eliminates floor and ceiling effects of the eight subscales and reduces the number of statistical comparisons (Ware et al., 1995). However, in the present study the use of the PCS was conceptually problematic because it contains a pain intensity scale (i.e., bodily pain), which would contaminate the relationship between the independent (i.e., numerical rating of pain intensity) and the dependent variable (i.e., physical health). Therefore, the Physical Functioning subscale, which measures the individual’s ability to perform in daily activities, was used in the present study as a measure of physical functioning. In accordance with standard practice for the SF-36, all scores were scaled to have a 0–100 range, a mean of 50, and a standard deviation of 10. High scores are interpreted as reflecting better health. Items in the SF-36 use various scale responses and response labels, so the reader is addressed to the validation paper for further information on item content (Ware and Sherbourne, 1992). The internal consistency of the SF-36 was good in the present study (0.69 < α < 0.93), consistent with previous reports (Alonso et al., 1998).

#### Rational/Irrational Beliefs

The short, Spanish version of the General Attitudes and Beliefs Scale (GABS-SV; Gonzalez et al., 1996) was used to evaluate participant’s tendency to appraise certain situations in a maladaptive manner (Burgess, 1986; DiGiuseppe et al., 1988). The questionnaire differentiates the four processes or styles of thinking proposed by Ellis (1962): demandingness (e.g., “I must have a pleasant, comfortable life most of the time”), catastrophizing (e.g., “It is a catastrophe to be hassled in life”), low frustration tolerance (LFT; e.g., “I cannot tolerate to fail at important tasks”), and self-downing (e.g., “I would be a worthless person if I achieved poorly at tasks that are important to me”). Each scale is composed of six items with response options ranging from 0 = *strongly disagree* to 4 = *strongly agree*. Thus, the maximum score for each scale is 24. All scales are bipolar, with lower scores reflecting rational thinking. The GABS-SV satisfies the recommendations for the assessment of beliefs: it distinguishes processes from content, evaluates cognition rather than behavior, and it does not include affective wording (Fulton et al., 2010). The internal consistency coefficients we obtained are comparable to those reported in previous research (Suso-Ribera et al., 2016). Specifically, estimates in our sample were 0.66 for demandingness, 0.90 for catastrophizing, 0.82 for LFT, and 0.77 for self-downing. The use of the GABS-SV as opposed to other well-established measures of rational/irrational thinking in the pain literature lies in the fact that only pain catastrophizing is frequently evaluated in chronic pain settings, while measures for the remaining forms of thinking are missing.

### Procedure

Participants in this study were recruited from a previous cross-sectional investigation conducted at the Vall d’Hebron Hospital in Barcelona from early 2013 to late 2015, in which the relationship between irrational beliefs and health status was investigated in a sample of 492 patients (Suso-Ribera et al., 2016). Since the previous study was published, 3 new patients have been recruited, so the current cross-sectional sample is composed of 495 patients. Six months after this cross-sectional evaluation was finished, patients were contacted again to investigate the longitudinal role of irrational beliefs in the recovery of these patients. These longitudinal findings are the ones presented in the current investigation.

Eligibility criteria included experiencing chronic pain (recurrent pain for at least 3 months in duration), being over 18 years of age, and giving written consent to participate. From 2013 to 2015, the clinical history of patients programmed for a first consultation at the pain unit was reviewed to check the eligibility criteria of age and pain duration. Next, potential participants were approached by letter by the lead researcher, CSR, 2 weeks before patients had their first appointment at the pain unit. Patients were asked to return the completed questionnaires on the day of the first visit, so all baseline measures were completed before the onset of medical treatment. On the day of the first medical appointment, either a physician or the lead researcher, CSR, officially enrolled the participants by collecting the written informed consent and the questionnaires. Five months after this first appointment, patients were contacted again by letter, and 1 month later (i.e., 6 months after the first appointment) they returned the new set of completed questionnaires (follow-up assessment). The protocol was the same for both assessment points and included an information sheet, an informed consent document, and the questionnaires. To explore the correlation between changes in pain intensity and changes in health status, both constructs were assessed at baseline and follow-up. By contrast, to test the study hypothesis, irrational beliefs were only measured at baseline.

All patients who completed the baseline assessment (*n* = 495) were contacted again approximately 5 months after the first evaluation. Of these, 163 patients returned the completed questionnaires (32.9%). Reasons for discontinuation could be explored for some patients, but these could not be changed. These reasons mostly included hospital discharge, which resulted in decreased motivation to participate in the study or perceived difficulties in delivering the questionnaires back to us, as well as lack of time and motivation.

All patients received the recommended treatment according to published guidelines (Finnerup et al., 2005; Cruccu et al., 2007; Attal et al., 2010; Brix Finnerup et al., 2010). This included pharmacotherapy (analgesics, non-steroid anti-inflammatory drugs, anticonvulsants, antidepressants, and opioids), interventional treatments (injections, radiofrequency, intrathecal pump implants, and spinal cord stimulation), topic treatments (creams and patches), and non-invasive electrical stimulation (transcutaneous electrical nerve stimulation and iontophoresis). The goal of the present study is not to discuss the effectiveness of each treatment for pain, but to explore whether a psychological construct, namely rational/irrational thinking, can help understand why changes in pain intensity, if existent, are not unequivocally associated with improved physical functioning. Therefore, a more detailed description of treatments for pain is out of the scope of the present investigation.

The Ethics Review Committee of the Vall d’Hebron Hospital in Barcelona approved the present study and all its procedures.

### Statistical Analyses

Because a large subset of patients who responded to the baseline assessment did not respond to the second administration (*n* = 332), we compared their characteristics against those of patients who provided data for both measurements. We used a *t*-test for independent samples to compare their age, pain duration, pain intensity, health status, and levels of irrational thinking. Cohen’s *d* effect sizes are reported. Additionally, we performed a χ^2^ test to explore differences in sex. These results are important to discuss the generalizability of findings. Cronbach’s alphas will also be calculated for all the study measures to ensure the internal consistency of scores.

Next, paired-samples *t*-tests were performed to examine changes in pain-related outcomes and psychological variables after medical treatment. Again, Cohen’s *d* effect sizes are reported. We also investigated sex differences in study variables, which might be informative for the reader and help justify the need to include sex as a covariate in the regression analyses. Additionally, Pearson correlation coefficients were calculated to assess the relationship between changes in pain intensity and changes in health, as well as the bivariate associations between baseline measures. To facilitate the interpretation of results, change scores were computed differently for pain intensity and health outcomes. Because pain intensity was expected to decrease with treatment, the change score was calculated by subtracting from baseline score, the post-treatment rating. By contrast, the physical and mental health status were expected to increase with treatment, so changes in health outcomes were obtained by subtracting from the post-treatment rating, the baseline score. By doing this, positive values in any of the change variables can be interpreted in the same direction, that is, as evidence showing that pain and health status improved.

Finally, a series of hierarchical analyses were performed in order to explore the moderating role of irrational thinking in the relationship between changes in pain intensity and changes in health. In the moderation analyses, variables were centered before creating the interaction term. Age, sex, and pain duration were used as covariates due to their relationship with study variables (Park et al., 2016). In order to interpret the moderation, a probing *post hoc* analysis of single slopes was conducted when a significant moderating effect was found. Significance was set at the alpha level of 0.01 to reduce the risk of Type I errors. To ensure that multicollinearity and influential observations were not a problem in the sample, we calculated the variance inflation factor and the standardized DFBETA, which should be smaller than 2 and 1, respectively (Stevens, 2003). There was no missing data in the study (the questionnaires were revised with the participants when returned at the pain clinic and any missing information was completed by participants on site).

All analyses were computed using PASW Statistics 22 (IBM Corp., 2013).

## Results

### Sample Characteristics and Comparison Between Study Completers and Participants Who Dropped Out

As reported in Table 1, we compared the baseline demographic and clinical characteristics of patients who completed both assessments (*n*_1_ = 163) and patients who only provided data for the baseline evaluation (*n*_2_ = 332) by means of a Student’s *t*-test. No differences were revealed in any of the continuous variables, including age, pain duration, pain intensity, health status, or rational thinking (all *p* > 0.01). The χ^2^ test did not reveal sex differences between completers and non-completers either (62.0 and 64.2% of females in the sample of completers and non-completers, respectively; χ^2^ = 0.23, *p* = 0.634).

**Table 1:** Means, standard deviations, and statistical differences in baseline scores between completers (*n* = 163) and non-completers (*n* = 332).

	Completers Mean (*SD*)	Non-completers Mean (*SD*)	*t*	*p*	95% CI	*d*
Age	58.74 (14.28)	58.47 (14.59)	-0.20	0.842	-3.00, 2.44	0.02
Pain duration	5.30 (7.56)	6.30 (8.71)	1.25	0.213	-0.57, 2.56	0.12
Pain intensity	7.79 (1.60)	7.71 (1.70)	-0.49	0.623	-0.39, 0.23	0.05
**Health status**						
PF	32.70 (23.80)	32.63 (24.29)	-0.03	0.978	-4.61, 4.48	<0.01
MCS	40.90 (13.05)	38.72 (13.22)	-1.72	0.087	-4.67, 0.32	0.17
**Irrational beliefs**						
Demandingness	17.59 (3.64)	17.33 (4.05)	-0.70	0.484	-1.00, 0.47	0.07
Catastrophizing	11.76 (5.92)	12.10 (6.39)	0.56	0.577	-0.84, 1.51	0.06
LFT	12.02 (5.17)	12.59 (5.93)	1.041	0.298	-0.50, 1.64	0.10
Self-downing	6.07 (5.31)	6.95 (5.46)	-0.03	0.978	-4.61, 4.48	0.16


### Sex Differences in Study Variables

As reported in Table 2, we found sex differences in pain intensity (*M*_men_ = 7.32, *SD*_men_ = 1.80, *M*_women_ = 8.08, *SD*_women_ = 1.40, *t* = -3.00, *p* = 0.003; 95% CI = -1.25, -0.26, *d* = 0.47) and physical functioning (*M*_men_ = 38.93, *SD*_men_ = 23.15, *M*_women_ = 28.87, *SD*_women_ = 23.49, *t* = 2.67, *p* = 0.008; 95% CI = 2.62, 17.50, *d* = 0.17). Specifically, women reported having more intense pain and were less able to perform in their daily activities due to their health problems. Sex differences were not observed in age, pain duration, mental health, changes in pain intensity and health outcomes after medical treatment, and rational thinking (all *p* > 0.01).

**Table 2:** Sex differences in study variables.

	Men Mean (*SD*) *n* = 62	Women Mean (*SD*) *n* = 101	*t*	*p*	95% CI	*d*
Age	55.64 (14.80)	60.64 (13.68)	-2.20	0.030	-9.50, -0.50	0.35
Pain duration	4.80 (6.70)	5.61 (8.06)	-0.67	0.504	-3.23, 1.59	0.11
Pain intensity	7.32 (1.80)	8.08 (1.40)	-3.00	0.003	-1.25, -0.26	0.47
Change in pain intensity	0.71 (2.15)	0.55 (1.63)	0.52	0.603	-0.43, 0.74	0.08
**Health status**						
PF	32.93 (23.15)	28.87 (23.49)	2.67	0.008	2.62, 17.50	0.17
MCS	43.31 (12.69)	39.41 (13.10)	1.87	0.064	-0.22, 8.03	0.30
Change in PF	5.18 (16.45)	4.53 (14.39)	0.26	0.792	-4.20, 5.49	0.04
Change in the MCS	-1.12 (8.88)	-0.21 (11.77)	-0.52	0.602	-4.34, 2.52	0.09
**Irrational beliefs**						
Demandingness	17.32 (3.57)	17.75 (3.69)	-0.73	0.466	-1.59, 0.73	0.12
Catastrophizing	11.08 (5.51)	12.18 (6.15)	-1.15	0.253	-2.98, 0.79	0.19
LFT	11.94 (5.35)	12.08 (5.08)	-0.17	0.864	-1.80, 1.51	0.03
Self-downing	5.97 (4.87)	6.14 (5.59)	-0.20	0.843	-1.87, 1.47	0.03


### Changes in Pain and Health Outcomes and Bivariate Associations Between Baseline Scores and Change Scores

Table 3 shows the mean-level differences in study outcomes (pain intensity and health status) after 6 months of medical treatment and the correlations between baseline scores. On average, pre-treatment pain reports fell within the moderate-to-severe range (Jensen et al., 2001a).

**Table 3:** Mean-level changes in pain intensity and health after 6 months of medical treatment and Pearson correlations between study variables.

	Mean (*SD*) baseline	Mean (*SD*) 6 months	*t*	95% CI	*d*	Pearson correlations between baseline scores					
						
						2	3	4	5	6	7
1. Pain intensity	7.79 (1.60)	7.18 (2.13)	4.25*	0.33, 0.90	0.32	-0.56*	-0.32*	0.06	-0.01	-0.04	-0.01
**Health status**											
2. PF	32.70 (23.80)	37.48 (26.23)	4.02*	4.43, 7.12	0.19		0.37*	-0.15	0.12	-0.03	-0.06
3. MCS	40.90 (13.05)	40.34 (13.05)	-0.66	-2.22, 1.11	0.04			-0.19	-0.46*	-0.46*	-0.44*
**Irrational beliefs**											
4. Demandingness	17.59 (3.64)								0.44*	0.41*	0.08
5. Catastrophizing	11.76 (5.92)									0.75*	0.60*
6. LFT	12.02 (5.17)										0.58*
7. Self-downing	6.07 (5.31)										


*PF, physical functioning; MCS, mental composite score; LFT, low frustration tolerance. ^∗^*p* < 0.001.*

Regarding changes at the mean-level, there was a significant reduction in pain intensity (*t* = 4.25, *p* < 0.001; 95% CI = 0.33, 0.90) and an increase in physical functioning ratings (*t* = 4.02, *p* < 0.001; 95% CI = 2.43, 7.12) after the intervention. Changes in pain intensity and physical health were between small and medium (*d* = 0.32 and *d* = 0.19, respectively). There were no significant changes in mental health at the group level (*t* = -0.66, *p* = 0.511; 95% CI = -2.22, 1.11).

The Pearson correlations indicated that pain intensity was significantly associated with poorer physical functioning (*r* = -0.56, *p* < 0.001) and mental health status (*r* = -0.32, *p* < 0.001). Irrational forms of thinking, were generally strongly intercorrelated and significantly associated with poorer mental health (Pearson correlation coefficients ranged from -0.44 to -0.46, except for demandingness). Irrational beliefs did not correlate with pain intensity and physical functioning.

Additionally, the bivariate analyses revealed that changes in pain intensity were moderately associated with changes in physical health status (*r* = 0.42, *p* < 0.001) and modestly correlated with changes in mental health (*r* = 0.20, *p* = 0.010).

### Moderation of Rational/Irrational Thinking

**FIGURE 1:**
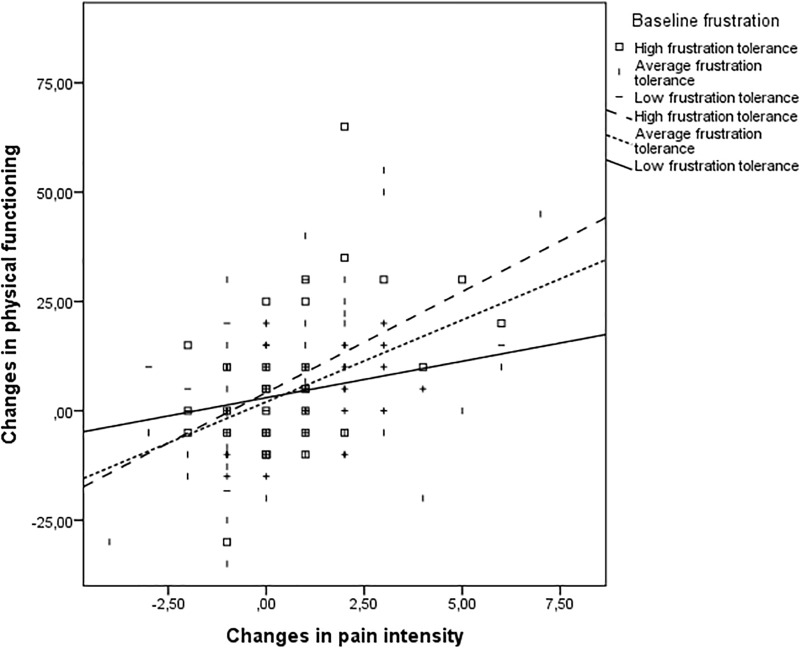
The moderating effect of low frustration tolerance in the relationship between changes in pain and changes in physical health after 6 months of medical treatment. Positive values in the change scores represent an improvement in the outcome (i.e., a reduction in pain ratings and increased physical functioning scores).

We explored whether irrational forms of thinking moderated the relationship between changes in pain intensity and changes in health status, with an emphasis on physical functioning as this was the measure of health status that revealed changes after the treatment. As reported in Table 4, baseline LFT moderated the relationship between changes in pain intensity and changes in physical functioning (β = -0.19, *t* = -2.67, *p* = 0.008; 95% CI = -0.52, -0.08). The negative beta coefficient in the interaction between LFT and changes in pain intensity indicates that LFT reduced the contribution of changes in pain intensity on changes in physical health status. A probing *post hoc* analysis and a graphical representation were performed to help interpret this finding (Figure 1). Simple slopes were calculated at ±1 *SD* from the mean of LFT and changes in pain intensity. At high levels of LFT (*M* = 17), changes in pain intensity were not related to changes in physical health (*r* = 0.28, *p* = 0.078, *n* = 41). Conversely, at low levels of LFT (*M* = 7) the relationship between changes in pain intensity and changes in physical health was moderate and significant (*r* = 0.47, *p* = 0.006, *n* = 32). Similarly, as reflected in Figure 1, the strength of the correlation between changes in pain intensity and changes in physical functioning increased with frustration tolerance. In other words, high frustration tolerance operated in favor of change after treatment (i.e., synergistic additive effect).

**Table 4:** Moderation of frustration tolerance in the relationship between changes in pain intensity and changes in physical functioning.

DV: change in the PCS		*b*	β	CI (95%)	*t*	*p*	*R*^2^ change	*F* change	*p*
1	Covariates						0.015	0.78	0.506
	Age	-0.09	-0.09	-0.25, 0.06	-1.17	0.053			
	Sex	-0.30	-0.01	-4.74, 4.15	-0.13	0.245			
	Pain duration	-0.16	-0.08	-0.45, 0.13	-1.07	0.895			
2	Change in pain intensity	3.36	0.41	2.20, 4.52	5.73	<0.001	0.171	33.14	<0.001
3	LFT baseline	-0.23	-0.08	-0.65, 0.19	-1.07	0.285	0.002	0.43	0.514
4	LFT × change in pain	-0.30	-0.19	-0.52, -0.08	-2.67	0.008	0.036	7.15	0.008


*PCS, Physical Composite Score; LFT, low frustration tolerance. Standardized (β) and unstandardized (*b*) betas refer to the final block of the regression. *R*^*2*^ change is unadjusted. Change scores were obtained by subtracting from baseline score, the post-treatment rating. Thus, positive change scores reflect a decrease in ratings after treatment (i.e., a reduction in pain intensity and physical health status).*

The remaining moderation effects were not significant, that is, the moderation of demandingness in the relationship between pain intensity and both physical functioning (β = 0.02, *t* = 0.29, *p* = 0.775; 95% CI = -0.20, 0.27) and mental health (β = -0.01, *t* = -0.11, *p* = 0.916; 95% CI = -0.19, 0.18), the moderation of catastrophizing in the relationship between pain intensity and both physical functioning (β = -0.10, *t* = -1.43, *p* = 0.154; 95% CI = -0.33, 0.05) and mental health (β = 0.05, *t* = 0.58, *p* = 0.564; 95% CI = -0.11, 0.20), the moderation of LFT in the relationship between pain intensity and mental health (β = 0.05, *t* = 0.61, *p* = 0.543; 95% CI = -0.12, 0.23), and the moderation of self-downing in the relationship between pain intensity and both physical functioning (β = 0.02, *t* = 0.27, *p* = 0.788; 95% CI = -0.21, 0.27) and mental health (β = 0.01, *t* = 0.15, *p* = 0.880; 95% CI = -0.17, 0.20).

No problems of data fit were detected. Specifically, the variance inflation factor was lower than 2 for all predictors, suggesting no problem of multicollinearity. The standardized DFBETA was smaller than 1 for all cases, so no problems of influential observations were detected either.

## Discussion

The aim of this study was to investigate the role of rational and irrational beliefs in the evolution of pain intensity and physical functioning and mental health after 6 months of medical treatment. Previous research had shown that pain reduction efforts do not necessarily result in improved physical health status (Skljarevski et al., 2010), as correlations between changes in pain intensity and changes in physical disability tend to be modest (Ohrbach and Dworkin, 1998; Sullivan et al., 2008; Menezes Costa et al., 2011). The current study extends previous findings revealing that psychological factors of the patient, namely LFT, can moderate the relationship between changes in pain intensity and changes in physical health. This might partly explain why reduced pain levels do not unequivocally lead to improved physical functioning.

Contrary to our expectations, only frustration tolerance emerged as a significant moderator in the present investigation. Catastrophizing and, to a lesser extent, demandingness and self-downing or self-criticism have been previously associated with pain outcomes (Stroud et al., 2000; Hadjistavropoulos et al., 2007; Okifuji and Turk, 2015; Ramírez-Maestre et al., 2017). Also importantly, these forms of thinking are moderately associated with each other (Suso-Ribera et al., 2016), which would justify our study hypotheses (i.e., that all of them would emerge as moderators). While acknowledging the similarities between all the aforementioned forms of thinking, in the next lines we will discuss specific features of frustration tolerance that might have influenced the present study findings. Frustration tolerance is a belief related to the appraisal of situations as being either unbearable (e.g., “I can’t deal with the difficulties life puts me through”) in its irrational pole or tolerable in its rational pole (e.g., “I can tolerate the difficulties life puts me through”) (Suso-Ribera et al., 2016). Thus, different to demandingness, catastrophizing, and self-downing, frustration tolerance taps into aspects of acceptance of reality, regardless of actual efforts to change that reality (Harrington, 2011). In fact, the similarity between acceptance, a construct that is well established in the chronic pain literature (McCracken et al., 2010), and frustration tolerance has been discussed in previous research (Harrington et al., 2007) and becomes evident when comparing items in the GABS-SV (e.g., “some situations are displeasing and uncomfortable, but I can still function despite them”; David et al., 2010) and items used to assess acceptance of pain (e.g., “I am getting on with the business of living no matter what my level of pain is”; Wicksell et al., 2009). Considering the amount of research showing the importance of acceptance in the context of pain, it is possible that low acceptance of reality, as reflected by high frustration intolerance scores, represents a key distinctive feature of frustration intolerance compared to other forms of thinking, which in turn helps to understand why only frustration tolerance emerged as a significant moderator in the pain-to-health relationship.

In addition to the described differences between frustration tolerance and the remaining forms of thinking, it is also important to note that past pain research has mostly investigated linear associations between thinking styles and outcomes, which are not necessarily generalizable to interaction effects (i.e., moderation). For instance, while pain catastrophizing has been reliably associated with numerous pain-related variables, its moderating role in predicting treatment efficacy (i.e., for whom treatment is more effective) has little support (Wertli et al., 2014), thus indicating that pain interventions will be similarly effective irrespective of baseline levels of pain catastrophizing. By contrast, there is evidence to suggest that acceptance, in the form of psychological flexibility, might explain differential responses to pain interventions (Probst et al., 2018). Consistent with the aforementioned studies, the present investigation revealed that psychological factors that are linearly related to pain outcomes, such as catastrophizing, might not necessarily moderate the effectiveness of interventions and provided further support for the importance of psychological constructs that tap into acceptance of reality (i.e., frustration tolerance) when predicting response to treatment in pain settings. These results should be interpreted as showing that pain treatment effectiveness will be comparable irrespective of baseline catastrophizing, demandingness, or self-downing characteristics of individuals. Additionally, they indicate that the patients’ tendency to tolerate discomforting events, such as experiencing pain, will be key in the progression of physical functioning after a medical intervention, maybe because some discomfort (i.e., pain) will still be experienced despite the reduction in pain levels. In other words, it is possible that being open to experience discomfort is more important than being realistic about future outcomes (i.e., low catastrophizing), non-demanding with reality, and self-compassionate (i.e., low self-downing) when it comes to making the most out of medical treatment for pain because some discomfort is likely to be present even if pain intensity is reduced with treatment. While these findings are in line with some previous similar research exploring the moderating role of pain catastrophizing and psychological flexibility in response to treatment (Wertli et al., 2014; Probst et al., 2018), it is important to note that that the present is the first investigation to explore the moderating role of rational thinking in the pain-to-health relationship after medical treatment and one of the first investigations to include all forms of irrational thinking in the same investigation in pain settings, so the reason why moderation only occurred for frustration tolerance and not for the remaining rational beliefs remains speculative at this stage and replication will be needed.

While acknowledging the previous limitation in the conclusions that can be drawn for the present study findings, past research has also shown that the belief that discomforting events cannot be tolerated boosts the negative impact of stressful situations on functioning (Harrington, 2011). By contrast, the belief that difficulties are challenges that can be dealt with is frequent found to be a source of resilience in the face of demanding situations (Esteve et al., 2007; Ramírez-Maestre et al., 2012). This relationship between thinking and outcomes is fundamental to understand how CBT conceptualizes the individuals’ functioning. CBT states that people’s behavior and emotional states are largely explained by how situations are experienced (Clark and Beck, 2010). Thus, according to this approach, irrational forms of thinking (e.g., catastrophizing about an event) would shape and bias information processing, ultimately leading to maladaptive emotional and behavioral reactions. Indeed, there is research to indicate that a change in irrational thinking is a mechanism explaining the effectiveness of CBT on depression (Cristea et al., 2015). This study evidenced that patients who presented a high frustration tolerance profile were more likely to obtain improvements in physical functioning proportional to the reduction in pain levels, which would support the practice of cognitive flexibility in CBT to increase the tolerance to frustration of these patients. Additionally, several forms of irrational thinking (i.e., catastrophizing, low frustration tolerance, and self-downing) were associated with poor mental health status cross-sectionally, which would provide further support for the important role of thought patterns in understanding emotional states. Only demandingness, which has already been argued to play a modest role when compared with the remaining irrational forms of thinking (Kelly et al., 1998; Suso-Ribera et al., 2016), was not related to mental health.

The moderation of pain-to-health associations after medical treatment is a key finding in the present investigation. The idea that psychological factors can act as moderators of treatment efficacy is not new. In fact, there is an increasing body of research supporting the role of psychological factors as moderators of the effectiveness of psychological interventions (Turner et al., 2007; Miles et al., 2011; Skinner et al., 2012). However, to the best our knowledge, this is the first study to demonstrate that psychological factors (i.e., frustration tolerance) can also be significant moderators of the effectiveness of medical treatments in pain settings. Specifically, our results indicate that the secondary gains of the intervention (i.e., improved physical functioning as a result of a reduction in pain levels) are higher when individuals present high frustration tolerance. There may be different mechanisms through which frustration tolerance influences the relationship between changes in pain intensity and changes in physical disability. One possibility is that the negativity of frustration intolerance hinders pain reduction efforts by distorting the perception of physical functionality. Congruent with this idea, one study revealed that depressed patients underestimate their objective levels of physical activity (Huijnen et al., 2010). An ingrained negative belief (e.g., “I can’t deal with physical challenges, such as climbing stairs”) might help create a biased perception that one is physically impaired, which might remain unaltered irrespective of pain reduction efforts. By contrast, a more positive, accepting appraisal of difficulties (e.g., “I can tolerate the pain when doing things that are important to me”) is known to lead to better physical performance (Vowles et al., 2011). Another possibility is that the belief that one cannot manage difficult situations leads to lower mood and, ultimately, to behavioral avoidance, thus contributing to physical disability. Supporting this hypothesis, frustration intolerance has been associated with depressed mood (Buschmann et al., 2018), poor mental health (Suso-Ribera et al., 2016), and low self-esteem (Stephenson et al., 2017). Depressed individuals are, in turn, less active physically (Schuch et al., 2017). Thus, it is possible that frustration-tolerant patients benefit more from the reduction of pain intensity because they present higher mood and remain physically more active. Both hypotheses remain merely speculative at this point.

Sample size was one of the strengths of the present study. Previously reported longitudinal investigations in pain settings have been generally small (i.e., between 40 and 70; for a review, see Jensen et al., 2011), which should make the present work findings relatively robust. However, there are of course a number of limitations in this investigation. Although we explored a set of important psychological factors in the chronic pain literature, especially catastrophizing, the list is far from complete. It is possible, therefore, that other variables frequently considered in pain settings (i.e., acceptance, fear, and perceived injustice) may also moderate the effectiveness of medical interventions. Also in relation to the assessed constructs, it is important to note that all measures were obtained with self-report methods. While this is a frequent practice in pain and health research, it is also true that it is possible that shared method variance might have influenced the results, resulting in stronger associations between variables. At this stage, this remains uncertain for the present study findings. However, the fact that only frustration tolerance and not all rational beliefs were significant moderators in the study makes us think that there is something unique in frustration tolerance which cannot be attributable to shared method variance only. Also importantly, the dropout rate in the study was high (67%) and population was characterized by experiencing heterogeneous pain (mostly low back and neck pain), so the generalizability of findings should be taken with care. While acknowledging this, the sample characteristics in our study (i.e., pain intensity and health status) are comparable (within a 1 *SD* range) to those of other pain clinics (Keeley et al., 2008; Wetherell et al., 2011), which should make our results useful for a wide number of clinicians and researchers. An additional aspect that should be considered is that the cross-sectional findings with the present study data have been already been reported in previous research (Suso-Ribera et al., 2016). Consequently, we address the readers to the previously reported work for further interpretation of cross-sectional findings. Note, however, that the inclusion of longitudinal data is clearly new to the present investigation and represents the key aim of the present investigation, for which research questions are largely different from those published previously. Finally, it should be noted that health status can be influenced by many factors other than pain, so we cannot ensure that the physical and mental functioning of patients in our sample was only influenced by pain. To control for this, we used important covariates of health in the regression analyses (i.e., sex and age), but, drawing from existent literature (Cano et al., 2008; Edwards et al., 2016; Cano-García et al., 2017; Kaiser et al., 2017), other candidates surely exist (e. g., medication misuse, treatment modality, anxiety or depressive symptoms, social or family support, and satisfaction with treatment, among others). The fact that the moderation existed while controlling for some important covariates of patient health status should make the present study results robust, but the inclusion of a more comprehensive set of covariates would be desirable to provide further support for the robustness and generalizability of the findings.

While acknowledging the aforementioned shortcomings, we believe that the present study might have important clinical implications. Physical disability due to chronic pain is matter of public concern as the indirect costs of the disease associated with physical limitations (i.e., sick leave, compensations) exceed medical costs for chronic pain patients by a factor of five (Turk, 2002; Gaskin and Richard, 2012). Consequently, it is important to maximize the positive effects that a reduction in pain has on physical functioning so that return to work and daily functioning after an effective pain treatment are enhanced (Hanley et al., 2008; Fedoroff et al., 2013). Thus, the results of the present study may be important in the context of personalized interventions. Personalized therapy has emerged as a result of the heterogeneity of patients’ responses to medical (LeResche et al., 2015) and psychological (Broderick et al., 2016) treatments. The goal of personalized interventions is to detect characteristics of the patient (i.e., genes, personality styles) that explain differences in the effectiveness of interventions (Chapman et al., 2014). The ultimate goal of this approach is to optimize treatment by selecting the most appropriate intervention for each individual. Take, for example, a patient with reports of high pain and poor physical health, arguably due to pain levels. In that situation, one would expect that physical functioning would be improved by decreasing pain intensity. In the light of our findings, this is likely to happen when patients think rationally (i.e., they present high frustration tolerance levels). As opposed to that, a different approach might be needed with patients presenting a low frustration tolerance profile, as they appear to respond similarly to both a decrease and an increase in pain intensity (i.e., no change in physical functioning). Psychological interventions (i.e., CBT) addressing beliefs such as frustration tolerance may therefore be useful in such cases. In fact, the promotion of rational thinking with CBT has already been shown to have positive effects on various health problems, such as hypertensive asthma and breast cancer patients (David et al., 2010). There is also evidence that beliefs can be changed in chronic pain settings (Jensen et al., 2001b; Turner et al., 2007; Morley et al., 2008). In the light of our results, we would expect that, as soon a more rational form of thinking is adopted, the positive impact that a reduction of pain intensity has on physical functioning will be enhanced. Note, though, that the nature of the present study prevents us from drawing any causal conclusions, so results should be interpreted with caution.

In sum, our results lead us to recommend the assessment of frustration tolerance beliefs before starting pain reduction interventions in pain settings. By doing so, we could personalize treatments by offering psychological treatment (i.e., CBT) to patients scoring low in this form of thinking in conjunction or prior to their usual medical treatment. Further studies are needed to replicate the present study findings, as well as to test whether an early psychological intervention targeting maladaptive beliefs can indeed maximize the secondary gains of pain-reduction efforts (i.e., improved physical functioning).

## Ethics Statement

The Ethics Review Committee of the Vall d’Hebron Hospital in Barcelona approved the present study and all its procedures. The protocol was the same for both assessment points and included an information sheet, an informed consent document, and the questionnaires.

## Author Contributions

All authors made substantial contributions to this work–helped in data interpretation, reviewed and discussed the manuscript, and approved the final version of the manuscript after a number of revisions. CS-R, SS-V, and DG-P designed the study. CS-R and LC-G collaborated in data acquisition. CS-R, JO, and DG-P worked in data analysis. CS-R elaborated the first draft of the manuscript.

## Conflict of Interest Statement

The authors declare that the research was conducted in the absence of any commercial or financial relationships that could be construed as a potential conflict of interest.
